# Entropy and Negative Specific Heat of Doped Graphene: Topological Phase Transitions and Nernst’s Theorem Revisited

**DOI:** 10.3390/e26090771

**Published:** 2024-09-10

**Authors:** L. Palma-Chilla, Juan A. Lazzús, J. C. Flores

**Affiliations:** 1 Departamento de Física, Universidad de La Serena, Casilla 554, La Serena 1700000, Chile; 2 Instituto de Investigación Multidisciplinario en Ciencias y Tecnología, Universidad de La Serena, Casilla 554, La Serena 1700000, Chile; 3 Departamento de Física, FACI, Universidad de Tarapacá, Casilla 7-D, Arica 1000000, Chile

**Keywords:** graphene, phase transitions, Boltzmann’s entropy, laws of thermodynamics

## Abstract

This study explores the thermodynamic properties of doped graphene using an adapted electronic spectrum. We employed the one-electron tight-binding model to describe the hexagonal lattice structure. The dispersion relation for graphene is expressed in terms of the hopping energies using a compositional parameter that characterizes the different dopant atoms in the lattice. The focus of the investigation is on the impact of the compositions, specifically the presence of dopant atoms, on the energy spectrum, entropy, temperature, and specific heat of graphene. The numerical and analytical results reveal distinct thermodynamic behaviors influenced by the dopant composition, including topological transitions, inflection points in entropy, and specific heat divergences. In addition, the use of Boltzmann entropy and the revision of Nernst’s theorem for doped graphene are introduced as novel aspects.

## 1. Introduction

Graphene is an artificial material with highly peculiar physical properties, including mechanical, electronic, optical, and thermodynamic characteristics [1,2,3,4,5]. These properties have sparked research interest for nearly 20 years, leading to a mature understanding of graphene’s behavior. While initial studies primarily focused on its inherent attributes, recent investigations delve into the effects of different deformations. These deformations have unveiled a new frontier for exploring this two-dimensional (2D) material, impacting various aspects such as the Fermi energy, density of states, electronic conductivity, thermal expansion coefficients, Grüneisen parameters, phase transition from a semimetal to a metal, elastic modulus, and phonon frequency [6,7,8,9,10,11,12,13,14].

On the other hand, the intrinsic properties of graphene can be further tailored with doping, which involves introducing different atoms into the graphene lattice. Doping graphene with various elements can modulate its electronic behavior, making it suitable for a wide range of applications [15,16]. One of the most studied forms of doping is with heteroatoms such as boron and nitrogen [17,18]. Boron-doped graphene introduces p-type doping, which enhances hole conductivity [19]. This is due to boron’s lower electronegativity compared to carbon, creating additional electron acceptor sites within the graphene lattice [20]. Similarly, nitrogen-doped graphene exhibits n-type behavior, increasing electron concentration and improving its performance in applications like sensors and catalysis [17,18].

The influence of doping on graphene’s electronic structure and properties has been extensively investigated. Studies have shown that doping can significantly affect the density of states and the band structure of graphene, leading to changes in its conductivity and thermal properties [21,22]. Understanding these and other effects is crucial for optimizing doped graphene for specific applications, whether in electronics, energies, or others [23,24].

In graphene, the tight-binding model facilitates the exploration of deformations by adjusting the hopping parameters and altering the distances between carbon atoms in the hexagonal lattice [25,26,27]. In this study, we employ the nearest-neighbor tight-binding model to introduce a variation in one of the hopping parameters, assuming locally two different wave functions. This approach allows us to model dopant atoms within a graphene lattice along this specific direction. Through this method, we investigate the energy spectrum topology and thermodynamics of doped graphene by numerically solving their electronic Hamiltonian. This methodology is based on previous studies employing the Gibbs entropy of pristine graphene [28]. An innovative aspect introduced in this study pertains to the use of Boltzmann entropy to obtain the thermodynamics of graphene and the revision of Nernst’s theorem for this system. As far as we know, no comparable application exists for doped graphene as proposed here.

## 2. Doped Graphene and Entropy

**Figure 1:**
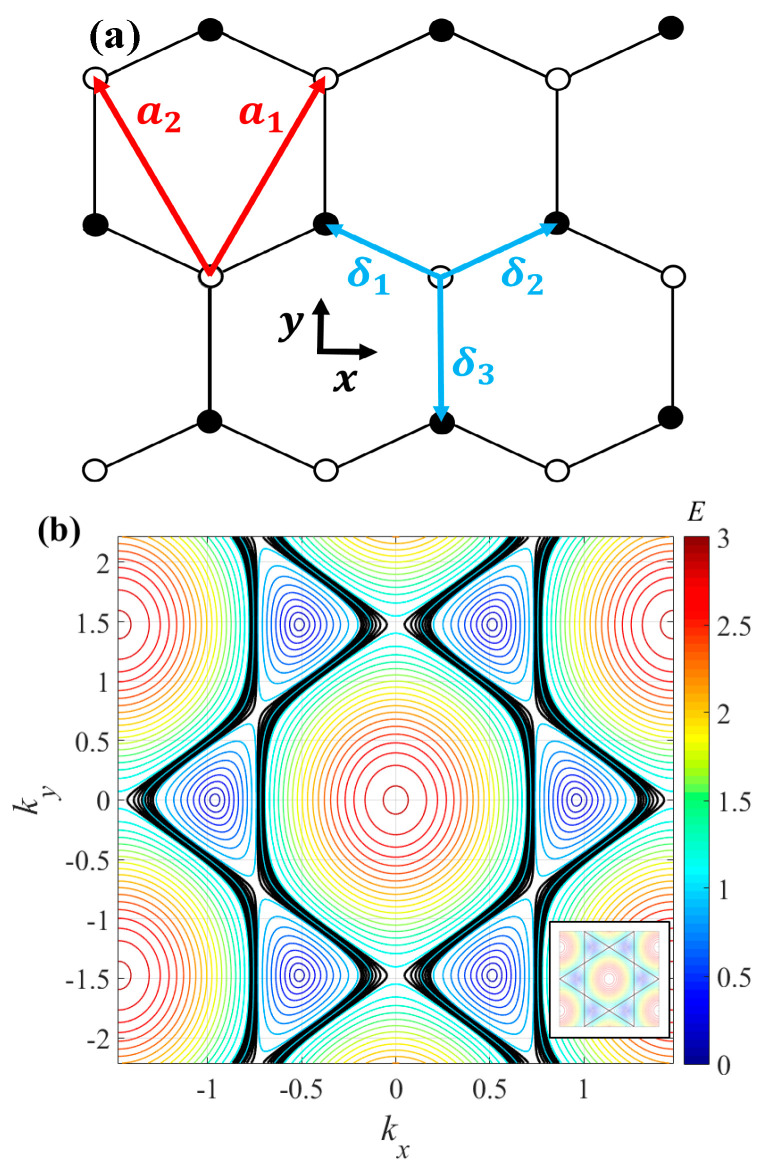
(**a**) Representation of the honeycomb lattice structure of a doped graphene, including the elementary vectors $a_{1}$ and $a_{2}$, and the nearest-neighbor vectors $\delta_{1}$, $\delta_{2}$, and $\delta_{3}$. White and black atoms represent the C and dopant atoms in the graphene lattice, respectively. (**b**) Graphical representation of the spectrum of doped graphene γ ~ 0.92 as a contour map of the energy dispersion obtained from Equation (3). As observed, the spectrum exhibits a double topological transition with energy levels that cross the entire cell (and the lattice) and are located between both topological transitions (note the black levels). In contrast, pristine graphene γ=1 exhibits only one topological transition when the energy levels pass from the six Dirac cones (lower energy levels) to only one central figure similar to a dome with upper energy levels (see the inner panel).

In the graphene tight-binding model, carbon atoms form a two-dimensional honeycomb lattice (see Figure 1a). This hexagonal structure can be characterized by a Bravais lattice whose vectors are defined as follows:

(1) $$a_{1}=\frac{a}{2}\;\left(\sqrt{3},3\right);\;\;\;\;\;\;a_{2}=\frac{a}{2}\;\left(-\sqrt{3},3\right)$$

where a is the honeycomb lattice parameter related to the distance between carbon atoms. It is important to note that the xy coordinate system has been chosen with the “x” axis in the zigzag direction and the “y” axis in the armchair direction of the graphene lattice [29,30,31,32]. We assume that the incorporation of substitutional (dopant) atoms into the graphene lattice does not change its 2D symmetry [33].

In addition, the vectors that connect a carbon atom with its nearest neighbors are defined as follows:

(2) $$\delta_{1}=\frac{a}{2}\;\left(\sqrt{3},1\right);\;\;\;\delta_{2}=\frac{a}{2}\;\left(-\sqrt{3},1\right);\;\;\;\;\delta_{3}=\frac{a}{2}\;\left(0,-1\right).$$

Therefore, by employing the tight-binding model for graphene and assuming the hopping parameters $t_{1}=t_{2}=t$ and $t_{3}=\gamma t$, the energy spectrum is obtained as follows:

(3) $$E=+t\sqrt{2+\gamma^{2}+4\gamma cos\left(\frac{\sqrt{3}}{2}ak_{x}\right)cos\left(\frac{3}{2}ak_{y}\right)+2cos\left(\sqrt{3}ak_{x}\right)}$$

where t (assumed unit) is the hopping energy term between the nearest carbon atoms. Note that this model represents the one-electron energy spectrum occupying the upper band and does not consider jumps from one band to another. The parameter γ is a compositional parameter that represents the ratio of hopping energies between the first neighbor atoms in the hexagonal graphene lattice ($\gamma =t_{3}/t$). We assume that varying atoms in the doped graphene lattice alter the topologies on the energy spectrum surface, resulting in distinct thermodynamic behaviors. Our approach applies to any type of doped graphene where its hopping energy ($t_{3}$) is slightly lower than that of pristine graphene (t~2.7 eV), which corresponds to cases where γ<1. Note that to maximize the number of cases where γ<1, the dopant and carbon atoms should be arranged in alternating positions in the hexagonal cell (see Figure 1a). On the other hand, Figure 1b shows a contour map of the energy graphene spectrum obtained with Equation (3). Thus, as examples of cases with γ<1, we consider two scenarios with a small difference in hopping energies: γ=~2.5 eV/2.7 eV≈0.92 and γ=~2.35 eV/2.7 eV≈0.86 (other examples of t values can be found in [22]). For contrast, the pristine case is γ=2.7 eV/2.7 eV=1.

The formal extreme case γ=0 corresponds to the full analytical spectrum $E=2t\left|cos\left(ak_{x}\sqrt{3}/2\right)\right|$ and is related to a one-dimensional motion for electrons. In this way, for smaller γ parameters, a one-dimensional motion predominance is expected (see Figure 1b).

To obtain the thermodynamic behavior of a doped graphene, we assume that the number of states $\Omega \left(E\right)$ [34,35,36,37,38] is directly proportional to the perimeter $P\left(E\right)$ generated by the intersection of the energy-cutting plane with the energy spectrum (from the electronic Hamiltonian) of the doped graphene. This fact considers that the degeneracy of the one-electron energy spectrum coincides directly with the degeneracy of the system. Note that for this analysis, the typical utilization of Gibbs entropy involves the consideration of geometrically closed lines (by using their area) within the contour map of the energy spectrum [28]. However, in our case, the preference for Boltzmann entropy arises from the presence of open contour lines in the energy spectrum of doped graphene (see Figure 1b). Then, Boltzmann’s entropy $S_{B}\left(E\right)$ was calculated through the following equation [34,35,36,37,38]:

(4) $$S_{B}\left(E\right)=k_{B}ln\left(P\left(E\right)\right)$$

where $k_{B}$ is the Boltzmann constant. In our approach, we equated the number of states to the size of the Fermi surface, specifically the two-dimensional Fermi surface. It is well known that there is an intrinsic connection between these properties [34,38]. Therefore, a computational method was developed to obtain $S_{B}\left(E\right)$ by applying the following algorithm:

(a) Numerically construct the energy surface of the doped graphene based on the electronic Hamiltonian equation.
(b) Select a specific energy level to establish the corresponding horizontal energy-cutting plane.
(c) Recognize the intersection points formed by the energy surface and the energy-cutting plane.
(d) Employ the identified intersection points to compute the perimeter P of the curve.

This algorithm iterates through the outlined steps within the full energy range of 0≤E≤3, calculating Boltzmann’s entropy for each 0.001 energy fraction of the spectrum and for each *γ* value studied.

**Figure 2:**
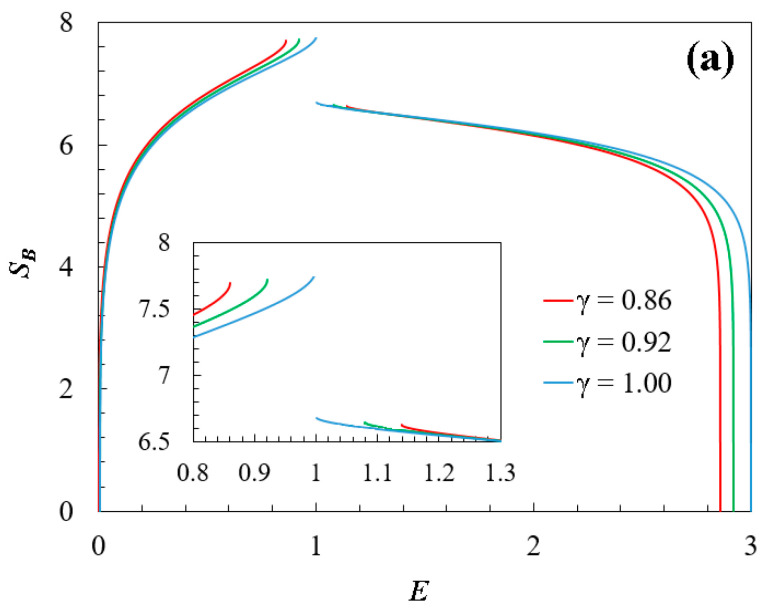

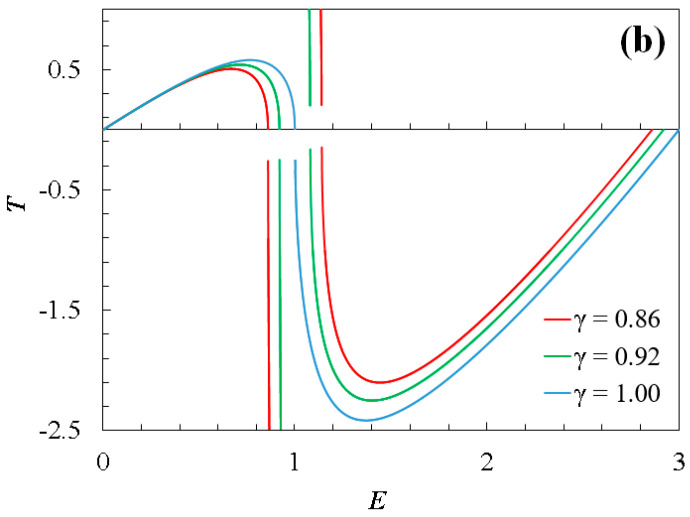
(**a**) Associated Boltzmann entropy $S_{B}(E)$ for the doped graphene spectrum as a function of dimensionless energy E. At a well-defined point, for any compositional parameter γ, the entropy exhibits a gap, and around this gap, there are two inflection points on the curve. For a better visualization, the inner panel shows a zoom of the gaps of the entropy curve. (**b**) Temperature $T\left(E\right)$ for the mentioned three cases as a function of dimensionless energy. Observe the existence of negative temperatures for “high-energy” ranges, and discontinuity in correspondence with the non-differentiable points of the entropy curve.

Figure 2a shows the Boltzmann entropy $S_{B}\left(E\right),$ for different values of the compositional parameter γ, as a function of the energy. Note that γ limits the support of the energy spectrum curve, that is, when γ<1, then $E_{max}<E_{max;pristine}$. In addition, there are gaps around E=1 for different values of this parameter, and the precise value of the gap depends on γ. Also, for any γ, there are two inflection points around where the second derivative of entropy is zero. These inflection points are directly related to specific heat divergences (next section). As observed, in every case, at low-energy conditions (E≪1), the entropy curves fit the equation $S_{B}/k_{B}=2ln\left(E\right)$, in consistency with the equipartition energy principle. Moreover, in this low-energy regime, doped graphene with γ ~ 0.92 obtains higher entropy values than the other two cases. Additionally, in this regime, the entropy of pristine graphene is lower than in the other cases. This is expected, as the pristine case has more translation symmetries than the others. It should be noted that the non-differentiable points on the entropy curve not only delineate the boundaries between positive and negative temperatures but also indicate the nearest topological transitions. Furthermore, in all cases studied, the entropy reached an asymptotic maximum value of approximately 7.75 units.

Given that the energy spectrum is bounded, as in the doped graphene analytical model from Equation (3), negative temperatures are expected [34,35,36,37,38]. Note that the physical meaning of negative temperature depends on the temperature of the thermal reservoir to which the system may be exposed. Using the thermodynamic relation $T={\left(𝜕E/𝜕S\right)}_{V}$, the temperature values for each doped graphene were numerically calculated. Figure 2b shows the temperature as a function of the dimensionless energy. It is interesting to see how compositional variation redefines the temperature spectrum. In the pristine case, the temperature is bounded exhibiting a global maximum and minimum (see blue line in Figure 2b). However, for cases with γ ~ 0.92 and ~0.86, any temperature is obtained (from +∞ to −∞) for any value of the energy spectrum, although with local maxima and minima (see green and red lines in Figure 2b). Thus, for the high-energy regime E>1, negative temperatures are obtained for different values of the compositional parameter. As observed, there are a relative maximum point and a relative minimum point for each temperature curve. In addition, at the low-energy regime, the temperature values exhibit a linear behavior fitting E~ 2T in accord with the energy equipartition principle and the Dirac equation. Also, in these regions, there is not a one-to-one correspondence between temperature and energy, which could indicate a particular type of phase transition. Note that the impact of γ shifts the maximum temperature value (or local maximum value for doped graphene) according to the curve T=0.52 γ+0.06 (with correlation coefficient $R^{2}=1$), while the minimum temperature (or local minimum value) is similarly shifted along the curve T=2.24 γ−0.18 with $R^{2}=1$. Additionally, the temperature curves exhibit a discontinuity that aligns with the non-differentiable points on the entropy curve and is potentially linked to the third law of thermodynamics (see Section 4). In this line, Nernst’s principle is numerically confirmed as E approaches 0 or $E_{max}$. That is, when T→0 then S→0 in the extreme points of the spectrum (see Figure 2a).

As a general remark regarding Figure 2, the compositional parameter (*γ*) influences the shape and features of the energy spectrum. These changes and topological transitions on the surface of the energy spectrum (see Figure 1b) affect the entropy and result in non-differentiable points in the entropy curve (see Figure 2a). These points can be associated with phase transitions or changes in the thermodynamic stability of the system. Additionally, the inflection points in the entropy curve correspond to maxima and minima in the temperature curve (see Figure 2b) and can be associated with possible divergences in the specific heat curve (see the next section).

## 3. Specific Heat Divergences

Specific heat values are determined by the thermodynamic relation $C={\left(𝜕E/𝜕T\right)}_{V}$, following the standard definition when no work occurs [34,35,36,37,38].

**Figure 3:**
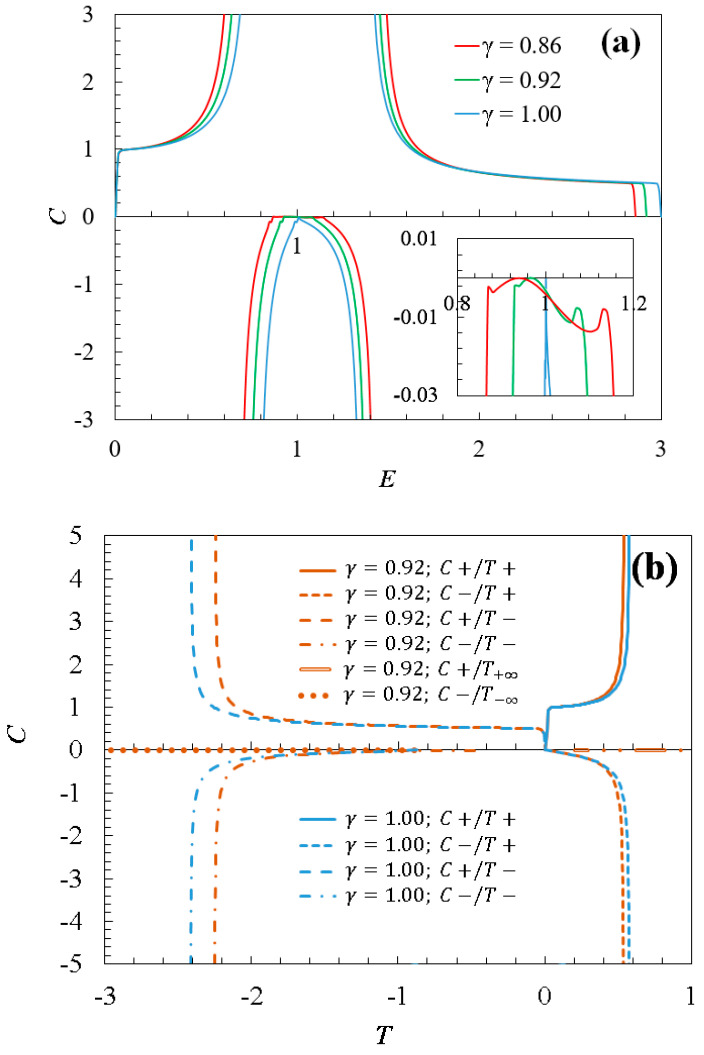
(**a**) Specific heat C for doped graphene (with γ ~ 0.92 and γ ~ 0.86) as a function of dimensionless energy E (the pristine case is also included). The inner panel shows a zoom picture around E~1 exhibiting three points with zero specific heat at T~0, one for each compositional parameter γ. (**b**) Specific heat as a function of temperature for doped graphene with γ ~ 0.92 compared to pristine graphene (details in the text).

Importantly in this study, the extreme points where 𝜕T/𝜕E=0 (see Figure 2b) are directly related to divergences of the specific heat (Figure 3a). These points define (topological) phase transitions since no order parameter seems to exist. Moreover, between two divergences for every C curve, there is a finite region with negative specific heat (see around E=1 in Figure 3a and its inset), which includes pristine graphene [28]. Note that the energy range (or domain) of this region is strongly related to the *γ* value. In fact, numerically, the domain of the C curve increases as the *γ* parameter decreases.

The expected phase transitions come from the simple fact that, by definition, heat variation δQ becomes directly related to the temperature variation δT through the following thermodynamic definition: δQ=CδT [35]. Consequently, for a given amount of heat δQ, when C=±∞, no temperature variations exist.

The specific heat divergences analytically come from the fundamental thermodynamic identity [36,37,38]:

(5) $$T^{2}C\frac{{𝜕}^{2}S}{{𝜕E}^{2}}=-1$$

Therefore, when the entropy possesses inflection points at finite temperature, as in the analyzed cases, the specific heat diverges. In fact, within the domain where the specific heat is negative (C<0), the second derivative of entropy is positive, indicating an association with a (relative) entropy minimum, a signature of instability. For γ=1, numerical calculations suggest C=0 according to the entropy inflection point at E=1, with two specific heat divergences at the numerical critical energy values E~0.76 and E~1.36. For γ<1, the doped graphene entropy also exhibits two inflection points, numerically approximated as γ~0.92→E~0.71 and E~1.40, and γ~0.86→E~0.66 and E~1.14, as well as two critical points in the energy spectrum (γ~0.92→E~0.92 and γ~0.86→E~0.86).

Figure 3a shows the specific heat C for different values of the compositional parameter (γ~0.86, γ~0.92, and γ=1) as a function of dimensionless thermodynamic energy E. On points where the temperature T=0, three points around E~1, and others in the borders (Figure 2), the specific heat becomes zero C=0, an expected consequence of Nernst-Planck’s theorem. Notice the presence of negative values for the specific heat and the existence of two divergences (for any value of γ). In the divergences, phase transitions are expected. The inner panel shows a zoom picture around E~1 showing two points with zero specific heat at T~0 for any parameter γ<1.

To clarify the thermodynamically stable and unstable regions for doped graphene by using specific heat, Figure 3b shows those values as a function of the temperature. Using the temperature-dependent specific heat C(T) of doped graphene, the representation includes two scenarios: one with γ~0.92 (as an example of the doped case) and another with γ=1 (for the pristine case). This graph depicts four curves for each type of graphene, illustrating their thermodynamic characteristics. The C+/T+ curve, where C values are positive when T is positive, exhibits stable behavior. On the other hand, the C−/T+, C+/T−, and C−/T− curves, with at least one parameter in the negative range, represent thermodynamically unstable curves. Here, the pristine case (γ=1, in blue lines) exhibits a combination of four regions defined by the sign of C and T. The curve C+/T+ is thermodynamically stable and corresponds to the 0≤E≤0.76 low-energy region in the energy spectrum. The rest of the curves are thermodynamically unstable and cover the higher-energy region of the spectrum ($0.76<E\leq E_{max,pristine}$). Also, the doped graphene (γ~0.92, in brown lines) exhibits a thermodynamically stable curve (C+/T+) corresponding to the low-energy region (0≤E≤0.71), while the unstable curves are in the higher-energy region ($0.71<E\leq E_{max}$). In other words, these graphs provide a clear visual representation of how specific heat varies with temperature for both pristine and doped graphene, highlighting the stability regions and distinguishing between stable and unstable curves in different energy regimes. In addition, this figure delves into a detailed analysis of the thermodynamic behavior of doped graphene, exploring the interplay between its energy spectrum, entropy, temperature, and specific heat. In this line, the divergence of specific heat that occurs when the second derivative of entropy is zero (Equation (5)) indicates, as mentioned, a phase transition. Note that between two specific heat divergences, there exists a region with negative specific heat (see Figure 3a). In this region, the second derivative of entropy is positive, indicating thermodynamic instability. The presence of negative specific heat is a notable thermodynamic feature, and its magnitude is influenced by the compositional parameter (γ). Different values of γ correspond to variations in the energy spectrum and, consequently, influence the specific heat behavior. This observation highlights the impact of dopant composition on the stability of doped graphene.

On the other hand, Figure 2 and Figure 3 also show evidence that for both pristine and doped graphene, there are three instances where the specific heat capacity becomes zero at absolute zero temperature (C=0 at T=0). These points are identified about the energy distribution extremes, with two of them aligning with T=0 and S=0 at the energy distribution extremes. The third point corresponds to the condition where entropy approaches a constant (S→const) at T=0, approximately at E~1 (see Figure 2a).

## 4. Nernst’s Theorem for Doped Graphene

Nernst’s theorem, or the third law of thermodynamics, states that for pure substances, when T→0 then S→0. Nevertheless, Planck supplements it by enouncing that the entropy for a pure substance, at T=0, is finite (“universal” residual entropy). An important and usual consequence of the third law is the thermodynamic limit for the specific heat [36,37,38]:

(6) $$\underset{T\to 0}{\lim}C=0$$

From our calculations (Figure 2a), and as said before, in the extreme energy points E=0 and $E=E_{\max}$, full Nernst’s theorem is operative for any compositional parameter 0≤γ≤1. Further, in the stable region (C≥0;T≥0), as the temperature decreases, the system tends to its lowest energy state E=0. Likewise, their entropy approaches zero (Figure 3a).

The interesting cases are the existence of additional energy points where T=0 around E~1 (see Figure 2b), and the entropy approaches non-zero constant values. This occurs at the energy regions where the (first) topological transition takes place (see Figure 2a, first branch). Additionally, in these (“internal”) zero-temperature points, there is numerical residual entropy depending on the values of the compositional parameter γ. Consequently, at least numerically, the residual entropy is not universal, since there is a mixture (impurities) of C and dopant atoms in the systems.

## 5. Conclusions

Based on the data analysis and analytical discussions in this study, the following conclusions were obtained:

The compositional parameter (*γ*) influences the energy spectrum of doped graphene, affecting its topological transitions and the thermodynamic stability of the system. Since dopant atoms within the graphene lattice impact the electronic structure, our results reveal several thermodynamic behaviors that are of particular interest in material science and related fields.

The Boltzmann entropy shows discontinuities and inflection points for different *γ* values. These points can be linked to (topological) phase transitions. Additionally, the temperature reveals negative values in the high-energy regime, while in the low-energy regime, the systems demonstrate consistency with the known thermodynamic fermionic theory in graphene.

Specific heat shows divergences associated with (topological) phase transitions for different *γ* values. Our numerical results connect specific heat, entropy, and temperature with thermodynamically stable/unstable regions in the energy spectrum. We observe regions of negative specific heat, which are influenced by the compositional parameter *γ*.

Nernst’s theorem was revisited through numerical calculations for doped graphene. In the thermodynamically stable region, as the temperature approaches absolute zero, the system tends towards its lowest energy state for the studied *γ* values. Additionally, an interesting observation includes extra internal energy points where the temperature reaches zero, corresponding to a scenario where entropy approaches a constant value that numerically depends on the compositional parameter *γ*.

Finally, the compositional parameter could be relevant for the substitution of other elements into the lattice, provided these substitutions are ordered, thereby lowering the symmetry of the 2D lattice. As a direction for future work, it would be valuable to investigate scenarios where the ratio of the dopant’s hopping energy to that of carbon exceeds *γ* > 1.

## Data Availability

The original contributions presented in the study are included in the article, further inquiries can be directed to the corresponding author.
